# Supplementary material for: Paternal and maternal psychiatric history and risk of preterm and early term birth: A nationwide study using Swedish registers
Source: PLoS Med. 2023 Jul 20;20(7):e1004256. doi: 10.1371/journal.pmed.1004256 (PMC10358938; doi:10.1371/journal.pmed.1004256)

Paternal and maternal psychiatric history and risk of preterm and early term birth: a nationwide study using Swedish registers

Weiyao Yin, Jonas F. Ludvigsson, Ulrika Åden, Kari Risnes, Martina Persson, Abraham Reichenberg, Michael E. Silverman, Eero Kajantie, Sven Sandin

List of contents

Supplementary Tables

Table A ICD codes for psychiatric disorders

Table B Percentiles of gestational age in days by parental psychiatric history.

Table C Parental psychiatric history and relative risk of preterm birth, adjusting for maternal smoking and BMI

Table D Parental psychiatric history before delivery and relative risk of very preterm birth (<32 weeks), moderate/late preterm birth (32-36 weeks) and early term birth (37-38 weeks) versus full term birth (≥39 weeks)

Table E Number of co-occurring psychiatric disorders in fathers and mothers and relative risk of preterm birth

Table F Parental psychiatric history and relative risk of preterm birth pairwise comparisons between exposure groups - father only, mother only and both parents

Table G Parental psychiatric history before delivery and relative risk of preterm birth, by spontaneous delivery and non-spontaneous delivery

Table H Parental psychiatric history before delivery and relative risk of preterm birth, by offspring sex

Table I Parental psychiatric history before delivery and relative risk of preterm birth in singletons

Table J Parental psychiatric history before delivery and relative risk of preterm birth, restricting to psychiatric disorders first being diagnosed prior to two years before conception

Table K Parental psychiatric history before delivery and relative risk of preterm birth, requiring at least two diagnoses over 30 days

Table L Parental psychiatric history before delivery and relative risk of preterm birth, restricting to births from 2005 onwards

Supplementary Figures

Fig A Flow diagram illustrating the identification of the study cohort

Fig B Density curve of gestational age (GA) distributions by parental psychiatric history

Fig C Cumulative distribution of years between birth of the child and last previous diagnosis in fathers and mothers

Table A ICD codes for psychiatric disorders

	ICD-10	ICD-9 (Swedish version)	ICD-8
Overall mental illness	F10-F99	295, 296, 29, 298, 299, 300, 301, 302, 303, 304, 305, 306, 307, 308, 309, 311, 312, 313, 314, 315, 316, 317, 318, 319	295, 296, 297, 298, 299, 300, 301, 302, 303, 304, 305, 306, 307, 308, 310, 311, 312, 313, 314, 315
**Psychoactive substance use**	F10-F19	303, 304, 305	303, 304
**Schizophrenia and other non-mood psychotic disorder**	F20–29	295, 297, 298C-X	295, 297, 298.2, 298.3, 298.9, 299
**Mood disorders**	F30-39	296, 298A, 298B, 300E, 311	296, 298.0, 298.1, 300.4
Depression	F32, F33, F341, F348, F349	296B, 300E, 311	296.0, 298.0, 300.4
Bipolar	F30, F31, F340	296CDE	296.1, 296.2, 296.3, 296.8
**Neurotic/behavioral disorders**	F40-F48, F50-59, F60-F69	300-302 (excl. 300E), 306, 307, 308, 309	300-302 (excl. 300.4), 305, 306, 307
Anxiety	F40, F41	300A, 300C	300.0,300.2
OCD	F42	300D	300.3
Stress-related disorder	F43	308, 309	
Somatoform disorder	F45	306	305, 306 (excl. 306.5)
Eating disorder	F50	307F	306.5
**Neurodevelopmental disorders, emotional and behavioral disorders of childhood origin and intellectual disability**	F70-F79, F80-F89, F90-98	299, 312-315, 317-319	308, 310-315
**Other psychiatric disorders**	F99	316	

Abbreviations. ICD: International Classification of Diseases; OCD: Obsessive Compulsive Disorder.

Note. Main or secondary diagnoses were registered in the *Swedish National Patient Register.*

Table B Percentiles of gestational age in days by parental psychiatric history

Psychiatric disorders	Parental psychiatric history	Percentiles of gestational age in days									
		1st	5th	10th	15th	20th	25th	50th	75th	90th	95th
Any psychiatric diagnosis	No P.hist	228	257	265	269	271	273	280	287	292	295
	Only paternal	224	255	264	268	271	273	280	287	292	295
	Only maternal	220	253	262	266	269	271	279	286	291	294
	Both parents	219	251	261	265	268	270	278	285	291	294
Substance use	No P.hist	227	256	265	268	271	273	280	287	292	295
	Only paternal	222	254	263	268	270	272	280	286	292	295
	Only maternal	216	252	262	266	269	271	279	286	291	294
	Both parents	220	250	261	265	268	270	278	285	291	294
Schizophrenia and other non-mood psychotic disorder	No P.hist	227	256	264	268	271	273	280	287	292	295
	Only paternal	220	253	262	267	270	272	280	286	292	295
	Only maternal	217	251	261	266	269	271	279	286	292	295
	Both parents	167	242	259	266	21	272	278	287	291	295
Mood disorders	No P.hist	227	256	265	268	271	273	280	287	292	295
	Only paternal	225	255	264	268	270	272	280	287	292	295
	Only maternal	220	253	261	266	268	270	278	285	291	294
	Both parents	216	250	260	264	267	270	277	285	291	294
Neurotic/behavioral disorders	No P.hist	228	256	265	268	271	273	280	287	292	295
	Only paternal	222	255	264	268	270	272	280	287	292	295
	Only maternal	219	253	262	266	268	270	278	285	291	294
	Both parents	216	249	260	264	267	270	278	285	291	294
Neurodevelopmental disorders	No P.hist	227	256	264	268	271	273	280	287	292	295
	Only paternal	222	253	262	267	270	272	280	286	292	295
	Only maternal	218	252	261	265	268	270	278	285	291	294
	Both parents	223	251	260	265	268	270	278	285	291	294

Abbreviations: P.hist: Psychiatric history.

Table C Parental psychiatric history and relative risk of preterm birth, adjusting for maternal smoking and BMI

Parental psychiatric history	Preterm (%)	Number of subjects	Model 1 RR (95% CI)	Model 2 RR (95% CI)	Model 3 RR (95% CI)
No P.hist	62,156 (5.50)	1,130,480	Reference	Reference	Reference
Only paternal	3,984 (5.97)	66,686	1.12 (1.09, 1.16)	1.05 (1.02, 1.08)	1.03 (1.00, 1.07) [1]
Only maternal	7,617 (6.86)	111,058	1.29 (1.26, 1.32)	1.26 (1.23, 1.29)	1.24 (1.21, 1.27)
Both parents	1,743 (7.89)	22,080	1.51 (1.44, 1.58)	1.36 (1.29, 1.42)	1.31 (1.25, 1.38)

Abbreviations. BMI: body mass index (kg/m^2); P.hist: Psychiatric history; RR: Relative risk; CI: confidence interval.

Note. RRs with 95% CIs were calculated using log binomial regression models with robust standard errors. Model 1: Adjusted for birth year by cubic natural splines with 5 knots; Model 2: Additionally adjusted for maternal and paternal education (< 9 years of primary education, 9 years of primary education, 1-2 years of secondary school education, 3 years of secondary school education, 1-2 years of postgraduate education, ≥ 3 years of postgraduate education, PhD education), income (modeled by ranks as natural cubic splines with five degrees of freedom) and age (as natural cubic splines with five degrees of freedom), all defined at delivery. Model 3: Additionally adjusted for maternal smoking during pregnancy (yes/no) and BMI at the first prenatal visit (<18.5 kg/m^2, 18.5-24.9 kg/m^2, 25-29.9 kg/m^2, ≥30 kg/m^2). [1] *P* value=0.036.

Table D Parental psychiatric history before delivery and relative risk of very preterm birth (<32 weeks), moderate/late preterm birth (32-36 weeks) and early term birth (37-38 weeks) versus full term birth (≥39 weeks)

Analysis groups	GA	Parental P.hist	Before full term (%)	Model 1 OR (95% CI)	Model 2 OR (95% CI)
Any P.hist	<32 week	No P.hist	10,510 (0.83)	Reference	Reference
		Only paternal	725 (0.99)	1.24 (1.15, 1.34)	1.06 (0.98, 1.15)
		Only maternal	1,366 (1.11)	1.51 (1.42, 1.60)	1.42 (1.34, 1.50)
		Both parents	292 (1.20)	1.69 (1.50, 1.91)	1.32 (1.17, 1.49)
	32-36 week	No P.hist	62,584 (4.93)	Reference	Reference
		Only paternal	3,872 (5.27)	1.12 (1.08, 1.16)	1.05 (1.01, 1.08)
		Only maternal	7,551 (6.16)	1.41 (1.38, 1.45)	1.37 (1.33, 1.40)
		Both parents	1,734 (7.14)	1.71 (1.62, 1.80)	1.53 (1.45, 1.61)
	37-38 week	No P.hist	230,854 (18.20)	Reference	Reference
		Only paternal	13,604 (18.51)	1.05 (1.03, 1.07)	1.02 (1.00, 1.04) [1]
		Only maternal	27,656 (22.56)	1.37 (1.35, 1.39)	1.35 (1.33, 1.37)
		Both parents	5,625 (23.15)	1.46 (1.42, 1.51)	1.38 (1.34, 1.43)
Substance use	<32 week	No P.hist	12,077 (0.85)	Reference	Reference
		Only paternal	373 (1.04)	1.26 (1.14, 1.40)	1.03 (0.93, 1.15)
		Only maternal	376 (1.29)	1.64 (1.48, 1.82)	1.45 (1.30, 1.61)
		Both parents	67 (1.23)	1.66 (1.30, 2.12)	1.20 (0.94, 1.53)
	32-36 week	No P.hist	71,501 (5.04)	Reference	Reference
		Only paternal	2,020 (5.63)	1.16 (1.11, 1.22)	1.05 (1.00, 1.10) [2]
		Only maternal	1,828 (6.25)	1.36 (1.29, 1.43)	1.26 (1.20, 1.33)
		Both parents	392 (7.22)	1.66 (1.49, 1.84)	1.41 (1.27, 1.57)
	37-38 week	No P.hist	263,310 (18.56)	Reference	Reference
		Only paternal	6,841 (19.07)	1.06 (1.03, 1.09)	1.00 (0.98, 1.03)
		Only maternal	6,284 (21.48)	1.25 (1.21, 1.28)	1.20 (1.17, 1.24)
		Both parents	1,304 (24.01)	1.47 (1.38, 1.57)	1.33 (1.25, 1.42)
Schizophrenia	<32 week	No P.hist	12,809 (0.86)	Reference	Reference
		Only paternal	35 (1.08)	1.30 (0.93, 1.81)	1.04 (0.75, 1.46)
		Only maternal	45 (1.37)	1.72 (1.28, 2.31)	1.52 (1.13, 2.05)
		Both parents	4 (4.21)	5.18 (1.93, 13.87)	3.91 (1.45, 10.50)
	32-36 week	No P.hist	75,317 (5.08)	Reference	Reference
		Only paternal	198 (6.12)	1.25 (1.08, 1.45)	1.14 (0.98, 1.32)
		Only maternal	221 (6.75)	1.44 (1.25, 1.65)	1.35 (1.18, 1.55)
		Both parents	5 (5.26)	1.05 (0.42, 2.60)	0.91 (0.37, 2.26)
	37-38 week	No P.hist	276,378 (18.65)	Reference	Reference
		Only paternal	632 (19.53)	1.09 (0.99, 1.18)	1.02 (0.93, 1.11)
		Only maternal	714 (21.81)	1.27 (1.16, 1.38)	1.18 (1.08, 1.28)
		Both parents	15 (15.79)	0.85 (0.49, 1.49)	0.76 (0.43, 1.32)
Mood disorders	<32 week	No P.hist	12,098 (0.85)	Reference	Reference

		Only paternal	189 (0.91)	1.11 (0.96, 1.28)	0.92 (0.79, 1.06)
		Only maternal	557 (1.13)	1.51 (1.38, 1.64)	1.40 (1.28, 1.53)
		Both parents	49 (1.34)	1.87 (1.41, 2.49)	1.43 (1.08, 1.91)
	32-36 week	No P.hist	71,040 (5.02)	Reference	Reference
		Only paternal	1,174 (5.66)	1.19 (1.12, 1.26)	1.10 (1.03, 1.16)
		Only maternal	3,257 (6.63)	1.52 (1.47, 1.58)	1.47 (1.41, 1.52)
		Both parents	270 (7.38)	1.79 (1.57, 2.03)	1.59 (1.40, 1.80)
	37-38 week	No P.hist	261,299 (18.46)	Reference	Reference
		Only paternal	3,908 (18.83)	1.05 (1.02, 1.09)	1.01 (0.98, 1.05)
		Only maternal	11,612 (23.62)	1.44 (1.41, 1.48)	1.40 (1.37, 1.43)
		Both parents	920 (25.14)	1.61 (1.49, 1.74)	1.51 (1.40, 1.63)
Neurotic/behavioral disorders	<32 week	No P.hist	11,271 (0.84)	Reference	Reference
		Only paternal	432 (1.05)	1.32 (1.19, 1.45)	1.14 (1.03, 1.25)
		Only maternal	1,065 (1.17)	1.58 (1.49, 1.69)	1.49 (1.39, 1.59)
		Both parents	125 (1.39)	1.99 (1.66, 2.37)	1.54 (1.28, 1.84)
	32-36 week	No P.hist	66,998 (4.97)	Reference	Reference
		Only paternal	2,228 (5.43)	1.15 (1.10, 1.20)	1.08 (1.03, 1.13)
		Only maternal	5,816 (6.37)	1.47 (1.43, 1.51)	1.42 (1.38, 1.46)
		Both parents	699 (7.76)	1.88 (1.74, 2.04)	1.68 (1.55, 1.82)
	37-38 week	No P.hist	246,364 (18.28)	Reference	Reference
		Only paternal	7,767 (18.93)	1.07 (1.05, 1.10)	1.04 (1.01, 1.07)
		Only maternal	21,451 (23.48)	1.44 (1.42, 1.47)	1.41 (1.39, 1.43)
		Both parents	2,157 (23.95)	1.54 (1.46, 1.62)	1.44 (1.37, 1.52)
Neurodevelopmental disorders	<32 week	No P.hist	12,547 (0.86)	Reference	Reference
		Only paternal	145 (1.08)	1.34 (1.13, 1.58)	1.03 (0.87, 1.22)
		Only maternal	183 (1.26)	1.66 (1.43, 1.92)	1.38 (1.19, 1.61)
		Both parents	18 (1.10)	1.46 (0.91, 2.32)	0.98 (0.61, 1.56)
	32-36 week	No P.hist	73,790 (5.06)	Reference	Reference
		Only paternal	829 (6.19)	1.32 (1.23, 1.41)	1.15 (1.07, 1.24)
		Only maternal	998 (6.87)	1.55 (1.46, 1.66)	1.41 (1.32, 1.50)
		Both parents	124 (7.56)	1.74 (1.45, 2.10)	1.42 (1.17, 1.71)
	37-38 week	No P.hist	271,389 (18.60)	Reference	Reference
		Only paternal	2,599 (19.40)	1.10 (1.05, 1.15)	1.05 (1.01, 1.10)
		Only maternal	3,392 (23.33)	1.41 (1.36, 1.47)	1.34 (1.29, 1.40)
		Both parents	359 (21.89)	1.33 (1.18, 1.50)	1.22 (1.08, 1.37)

Abbreviations. P.hist: Psychiatric history; GA: gestational age; OR: Odds ratio; Cl: confidence interval.

Note. ORs of gestational age <32 weeks, gestational age 32 to 36 weeks and gestational age 37-38 weeks, versus gestational age ≥39 weeks at delivery, with 95% confidence interval, using multi-nominal logistic regression models. Model 1 adjusted for birth year as cubic natural splines with 5 knots. Model 2: Additionally adjusted for maternal and paternal education (< 9 years of primary education, 9 years of primary education, 1-2 years of secondary school education, 3 years of secondary school education, 1-2 years of postgraduate education, ≥ 3 years of postgraduate education, PhD education), income (modeled by ranks as natural cubic splines with five degrees of freedom) and age (as natural cubic splines with five degrees of freedom), all defined at delivery. [1] *P* value=0.070. [2] *P* value=0.031.

Table E Number of co-occurring psychiatric disorders in fathers and mothers and relative risk of preterm birth

Number of co-occurring psychiatric disorders	Number of subjects	Preterm (%)	Model 1 RR (95% CI)	Model 2 RR (95% CI)
Paternal P.hist				
None	1,391,118	82,011 (5.90)	Reference	Reference
One category [1]	70,958	4,648 (6.55)	1.10 (1.07, 1.13)	1.04 (1.01, 1.07)
Two categories	18,540	1,293 (6.97)	1.15 (1.09, 1.21)	1.05 (0.99, 1.11)
≥Three categories	8,304	682 (8.21)	1.33 (1.23, 1.43)	1.17 (1.09, 1.26)
Maternal P.hist				
None	1,342,007	77,691 (5.79)	Reference	Reference
One category	100,356	7,120 (7.09)	1.25 (1.22, 1.28)	1.22 (1.19, 1.25)
Two categories	33,802	2,636 (7.80)	1.39 (1.34, 1.44)	1.34 (1.29, 1.39)
≥Three categories	12,755	1,187 (9.31)	1.65 (1.56, 1.74)	1.55 (1.47, 1.64)

Abbreviations. P.hist: Psychiatric history; RR: Relative risk; CI: confidence interval.

Note. RRs with 95% CIs were calculated using log binomial regression models with robust standard errors. Model 1: Adjusted for birth year by cubic natural splines with 5 knots and any psychiatric diagnosis among the opposite parent; Model 2: Additionally adjusted for maternal and paternal education (< 9 years of primary education, 9 years of primary education, 1-2 years of secondary school education, 3 years of secondary school education, 1-2 years of postgraduate education, ≥ 3 years of postgraduate education, PhD education), income (modelled by ranks as natural cubic splines with five degrees of freedom) and age (as natural cubic splines with five degrees of freedom), all defined at delivery. [1] Categories of psychiatric diagnoses: psychoactive substance use, schizophrenia spectrum, mood disorders, neurotic/behavioral disorders, neurodevelopmental disorders, and other psychiatric disorder (not otherwise specified).

Table F Parental psychiatric history and relative risk of preterm birth: Pairwise comparisons between exposure groups - father only, mother only and both parents

Analysis groups	Model 1 RR (95% CI)	Model 2 RR (95% CI)
Only maternal vs only paternal	1.17 (1.13, 1.21)	1.22 (1.17, 1.26)
Both parents vs only maternal	1.16 (1.11, 1.22)	1.07 (1.02, 1.12)
Both parents vs only paternal	1.36 (1.29, 1.43)	1.30 (1.24, 1.37)
	Preterm (%)	Number of subjects
Only paternal	4,597 (6.25)	73,500
Only maternal	8,917 (7.27)	122,611
Both parents	2,026 (8.34)	24,302
Neither parent	73,094 (5.76)	1,268,507

Abbreviations. P.hist: Psychiatric history; RR: Relative risk; CI: confidence interval.

Note. RRs with 95% CIs were calculated using log binomial regression models with robust standard errors. Model 1: Adjusted for birth year by cubic natural splines with 5 knots; Model 2: Additionally adjusted for maternal and paternal education (< 9 years of primary education, 9 years of primary education, 1-2 years of secondary school education, 3 years of secondary school education, 1-2 years of postgraduate education, ≥ 3 years of postgraduate education, PhD education), income (modeled by ranks as natural cubic splines with five degrees of freedom) and age (as natural cubic splines with five degrees of freedom), all defined at delivery.

Table G Parental psychiatric history before delivery and relative risk of preterm birth, by spontaneous delivery and non-spontaneous delivery

Subgroups	Number of subjects	Preterm (%)	Model 1 RR (95% CI)	Model 2 RR(95% CI)
Spontaneous delivery				
No P.hist	1,002,232	45,951 (4.58)	Reference	Reference
Only paternal	56,654	2,888 (5.10)	1.13 (1.09, 1.17)	1.06 (1.02, 1.10)
Only maternal	86,961	5,073 (5.83)	1.30 (1.26, 1.34)	1.26 (1.22, 1.30)
Both parents	17,107	1,196 (6.99)	1.57 (1.48, 1.66)	1.41 (1.33, 1.49)
Non-spontaneous delivery				
No P.hist	266,275	27,143 (10.19)	Reference	Reference
Only paternal	16,846	1,709 (10.14)	1.07 (1.02, 1.12)	1.00 (0.96, 1.05)
Only maternal	35,650	3,844 (10.78)	1.15 (1.12, 1.19)	1.12 (1.09, 1.16)
Both parents	7,195	830 (11.54)	1.28 (1.20, 1.37)	1.15 (1.08, 1.23)

Abbreviations. P.hist: Psychiatric history; RR: Relative risk; CI: confidence interval.

Note. RRs with 95% CIs were calculated using log binomial regression models with robust standard errors among spontaneous and non-spontaneous deliveries separately. Model 1: Adjusted for birth year by cubic natural splines with 5 knots; Model 2: Additionally adjusted for maternal and paternal education (< 9 years of primary education, 9 years of primary education, 1-2 years of secondary school education, 3 years of secondary school education, 1-2 years of postgraduate education, ≥ 3 years of postgraduate education, PhD education), income (modelled by ranks as natural cubic splines with five degrees of freedom) and age (as natural cubic splines with five degrees of freedom), all defined at delivery.

Table H Parental psychiatric history before delivery and relative risk of preterm birth, by offspring sex

Parental psychiatric history	Number of subjects	Preterm (%)	Model 1 RR (95% CI)	Model 2 RR (95% CI)
Male offspring				
No P.hist	653,129	39,265 (6.01)	Reference	Reference
Only paternal	37,723	2,481 (6.58)	1.12 (1.08, 1.17)	1.05 (1.01, 1.10)
Only maternal	63,104	4,765 (7.55)	1.30 (1.26, 1.34)	1.26 (1.22, 1.30)
Both parents	12,526	1,057 (8.44)	1.47 (1.39, 1.56)	1.32 (1.25, 1.41)
Female offspring				
No P.hist	615,378	33,829 (5.50)	Reference	Reference
Only paternal	35,777	2,116 (5.91)	1.11 (1.06, 1.16)	1.03 (0.99, 1.08)
Only maternal	59,507	4,152 (6.98)	1.32 (1.28, 1.36)	1.28 (1.24, 1.32)
Both parents	11,776	969 (8.23)	1.58 (1.48, 1.68)	1.40 (1.31, 1.49)

Abbreviations. P.hist: Psychiatric history; RR: Relative risk; CI: confidence interval.

Note. Interaction between parental psychiatric history before delivery and offspring sex was tested by Wald test and was not statistically significant (*P* value=0.42). RRs with 95% CIs were calculated using log binomial regression models with robust standard errors among male and female offspring separately. Model 1: Adjusted for birth year by cubic natural splines with 5 knots; Model 2: Additionally adjusted for maternal and paternal education (< 9 years of primary education, 9 years of primary education, 1-2 years of secondary school education, 3 years of secondary school education, 1-2 years of postgraduate education, ≥ 3 years of postgraduate education, PhD education), income (modelled by ranks as natural cubic splines with five degrees of freedom) and age (as natural cubic splines with five degrees of freedom), all defined at delivery.

Table I Parental psychiatric history before delivery and relative risk of preterm birth in singletons

Parental psychiatric history	Number of subjects	Preterm (%)	Model 1 RR (95% CI)	Model 2 RR (95% CI)
No P.hist	1,230,480	56,641 (4.60)	Reference	Reference
Only paternal	71,362	3,586 (5.03)	1.12 (1.08, 1.16)	1.03 (0.99, 1.06)
Only maternal	118,924	7,195 (6.05)	1.36 (1.32, 1.39)	1.29 (1.26, 1.32)
Both parents	23,610	1,684 (7.13)	1.62 (1.55, 1.70)	1.39 (1.32, 1.46)

Abbreviations. P.hist: Psychiatric history; RR: Relative risk; CI: confidence interval.

Note. RRs with 95% CIs were calculated using log binomial regression models with robust standard errors. Model 1: Adjusted for birth year by cubic natural splines with 5 knots; Model 2: Additionally adjusted for maternal and paternal education (< 9 years of primary education, 9 years of primary education, 1-2 years of secondary school education, 3 years of secondary school education, 1-2 years of postgraduate education, ≥ 3 years of postgraduate education, PhD education), income (modelled by ranks as natural cubic splines with five degrees of freedom) and age (as natural cubic splines with five degrees of freedom), all defined at delivery.

Table J Parental psychiatric history before delivery and relative risk of preterm birth, restricting to psychiatric disorders first being diagnosed prior to two years before conception

Parental psychiatric history	Number of subjects	Preterm (%)	Model 1 RR (95% CI)	Model 2 RR (95% CI)
No P.hist	1,309,789	75,978 (5.80)	Reference	Reference
Only paternal	63,532	4,106 (6.46)	1.15 (1.11,1.18)	1.07 (1.03,1.10)
Only maternal	100,054	7,262 (7.26)	1.30 (1.27,1.33)	1.25 (1.22,1.28)
Both parents	15,545	1,288 (8.29)	1.50 (1.42,1.58)	1.34 (1.27,1.41)

Abbreviations. P.hist: Psychiatric history; RR: Relative risk; CI: confidence interval.

Note. RRs with 95% CIs were calculated using log binomial regression models with robust standard errors. Model 1: Adjusted for birth year by cubic natural splines with 5 knots; Model 2: Additionally adjusted for maternal and paternal education (< 9 years of primary education, 9 years of primary education, 1-2 years of secondary school education, 3 years of secondary school education, 1-2 years of postgraduate education, ≥ 3 years of postgraduate education, PhD education), income (modelled by ranks as natural cubic splines with five degrees of freedom) and age (as natural cubic splines with five degrees of freedom), all defined at delivery.

Table K Parental psychiatric history before delivery and relative risk of preterm birth, requiring at least two diagnoses over 30 days

Parental psychiatric history	Number of subjects	Preterm (%)	Model 1 RR (95% CI)	Model 2 RR (95% CI)
No P.hist	1,382,731	80,515 (5.82)	Reference	Reference
Only paternal	33,551	2,267 (6.76)	1.20 (1.15,1.25)	1.09 (1.04,1.13)
Only maternal	63,072	5,006 (7.94)	1.42 (1.38,1.46)	1.37 (1.33,1.41)
Both parents	9,566	846 (8.84)	1.60 (1.50,1.70)	1.40 (1.31,1.50)

Abbreviations. P.hist: Psychiatric history; RR: Relative risk; CI: confidence interval.

Note. RRs with 95% CIs were calculated using log binomial regression models with robust standard errors. Model 1: Adjusted for birth year by cubic natural splines with 5 knots; Model 2: Additionally adjusted for maternal and paternal education (< 9 years of primary education, 9 years of primary education, 1-2 years of secondary school education, 3 years of secondary school education, 1-2 years of postgraduate education, ≥ 3 years of postgraduate education, PhD education), income (modelled by ranks as natural cubic splines with five degrees of freedom) and age (as natural cubic splines with five degrees of freedom), all defined at delivery.

Table L Parental psychiatric history before delivery and relative risk of preterm birth, restricting to births from 2005 onwards

Parental psychiatric history	Number of subjects	Preterm (%)	Model 1 RR (95% CI)	Model 2 RR (95% CI)
No P.hist	737,723	40,858 (5.54)	Reference	Reference
Only paternal	56,917	3,453 (6.07)	1.11 (1.07,1.15)	1.03 (1.00,1.07)[1]
Only maternal	101,454	7,235 (7.13)	1.31 (1.27,1.34)	1.27 (1.24,1.30)
Both parents	21,767	1,773 (8.15)	1.50 (1.44,1.57)	1.33 (1.27,1.40)

Abbreviations. P.hist: Psychiatric history; RR: Relative risk; CI: confidence interval.

Note. RRs with 95% CIs were calculated using log binomial regression models with robust standard errors. Model 1: Adjusted for birth year by cubic natural splines with three knots; Model 2: Additionally adjusted for maternal and paternal education (< 9 years of primary education, 9 years of primary education, 1-2 years of secondary school education, 3 years of secondary school education, 1-2 years of postgraduate education, ≥ 3 years of postgraduate education, PhD education), income (modelled by ranks as natural cubic splines with three degrees of freedom) and age (as natural cubic splines with three degrees of freedom), all defined at delivery. [1] *P* value =0.073.

Fig A Flow diagram illustrating the identification of the study cohort

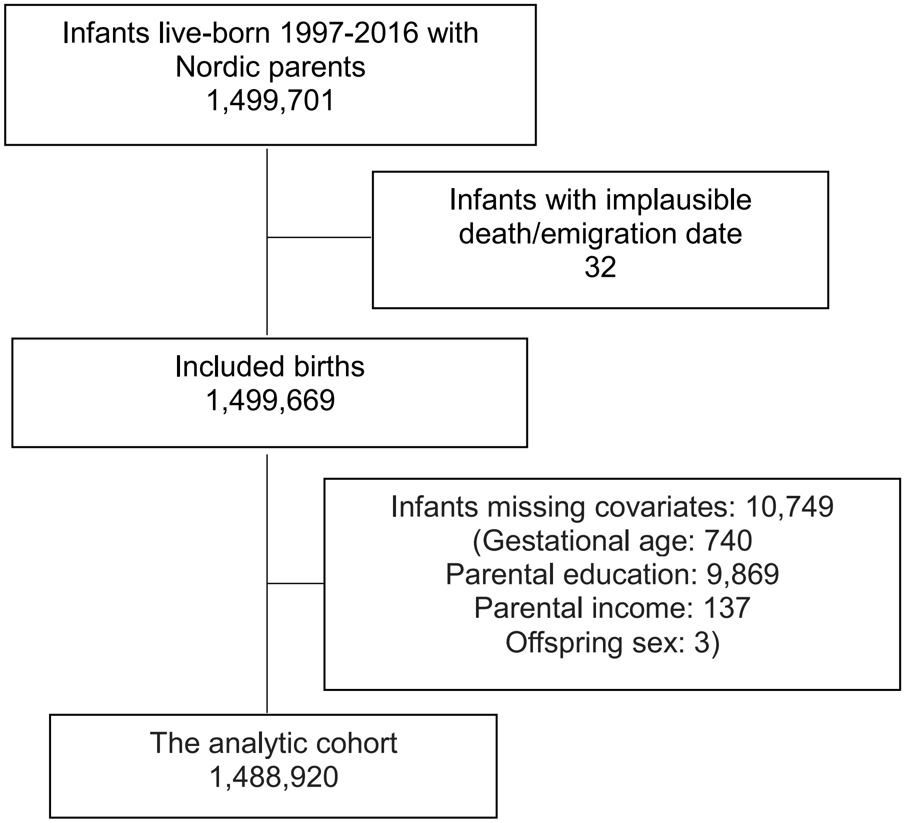

Note: Due to data protection, we only had access to date of birth by year and month, and assigned all birth dates as the first date of each month.

Fig B Density curve of gestational age (GA) distributions by parental psychiatric history

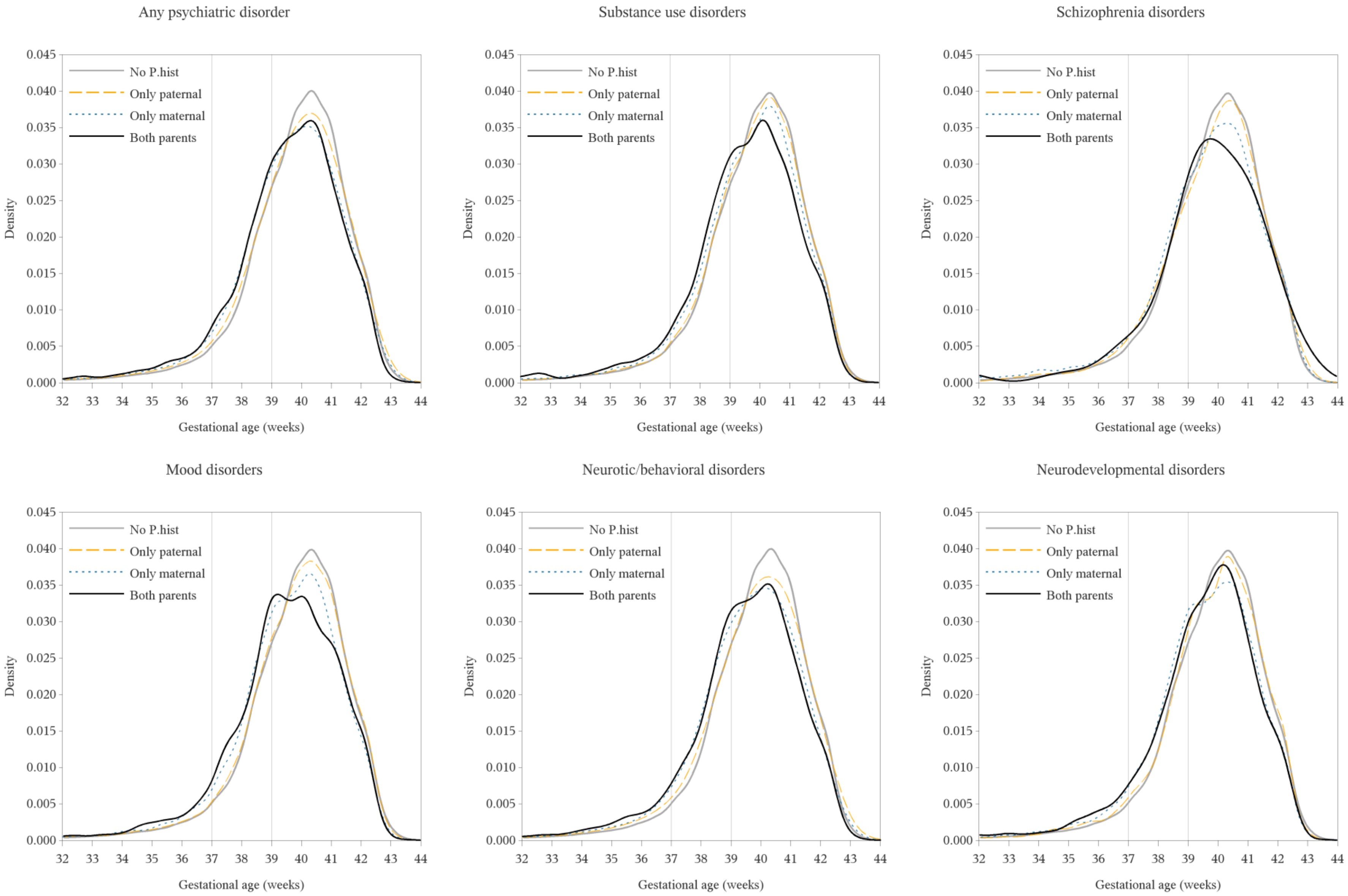

Abbreviations. GA: Gestational age.

Note. For each psychiatric diagnostic category, the GA distribution was described by the probability density function. The area under each curve is the same, but the distribution of the data may differ depending on differences in parental psychiatric history and by different psychiatric disorders.

Fig C Cumulative distribution of years between birth of the child and last previous diagnosis in fathers and mothers

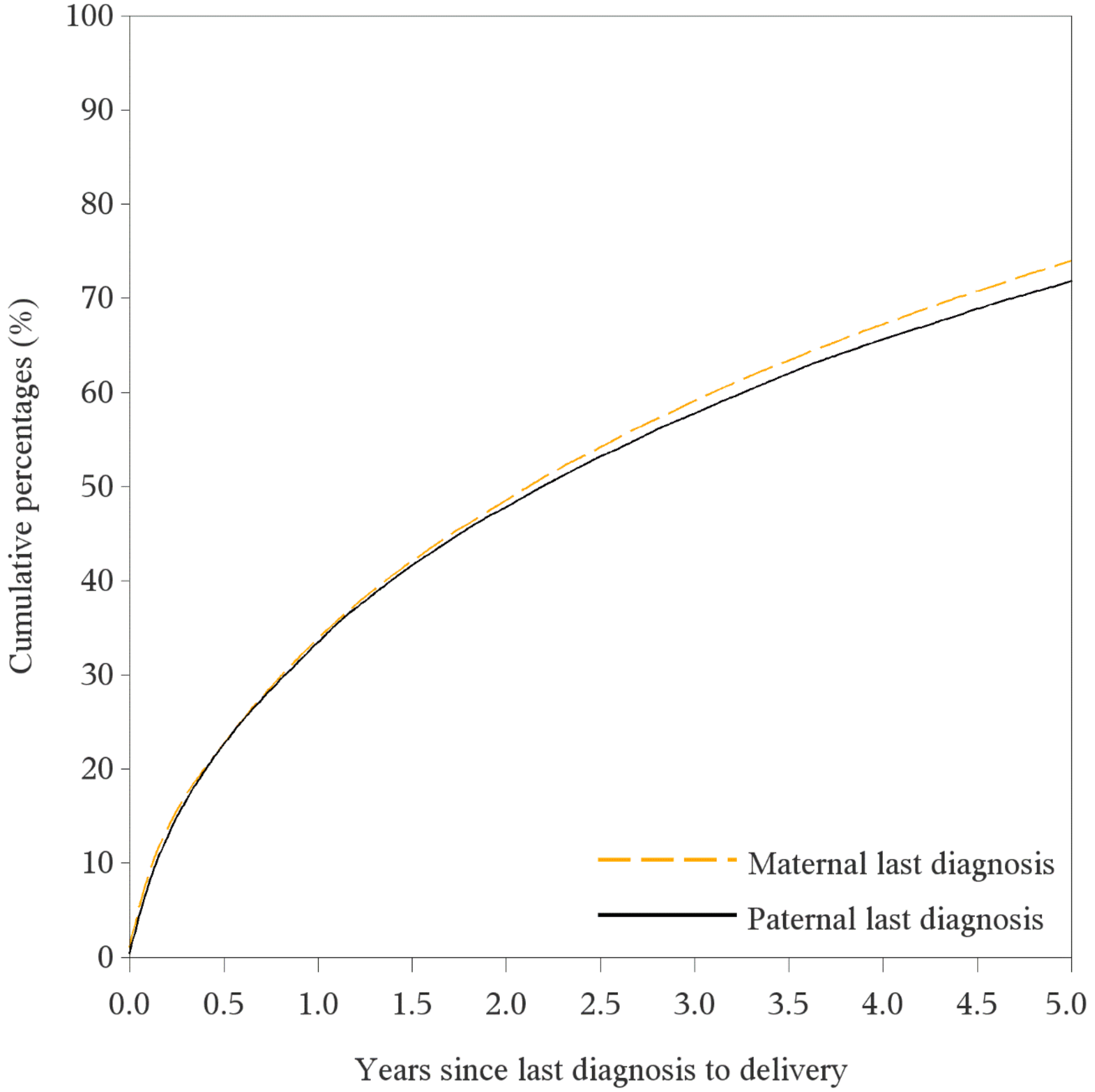

Supplement: S1 Appendix — Table A. ICD codes for psychiatric disorders. Table B. Percentiles of gestational age in days by parental psychiatric history. Table C. Parental psychiatric history and relative risk of preterm birth, adjusting for maternal smoking and BMI. Table D. Parental psychiatric history before delivery and relative risk of very preterm birth (<32 weeks), moderate/late preterm birth (32–36 weeks), and early term birth (37–38 weeks) vs. full term birth (≥39 weeks). Table E. Number of co-occurring psychiatric disorders in fathers and mothers and relative risk of preterm birth. Table F. Parental psychiatric history and relative risk of preterm birth pairwise comparisons between exposure groups—father only, mother only, and both parents. Table G. Parental psychiatric history before delivery and relative risk of preterm birth, by spontaneous delivery and non-spontaneous delivery. Table H. Parental psychiatric history before delivery and relative risk of preterm birth, by offspring sex. Table I. Parental psychiatric history before delivery and relative risk of preterm birth in singletons. Table J. Parental psychiatric history before delivery and relative risk of preterm birth, restricting to psychiatric disorders first being diagnosed 2 years before conception. Table K. Parental psychiatric history before delivery and relative risk of preterm birth, requiring at least 2 diagnoses over 30 days. Table L. Parental psychiatric history before delivery and relative risk of preterm birth, restricting to births from 2005 onwards. Fig A. Flow diagram illustrating the identification of the study cohort. Fig B. Density curve of gestational age (GA) distributions by parental psychiatric history. Fig C. Cumulative distribution of years between birth of the child and last previous diagnosis in fathers and mothers. (PDF) [file pmed.1004256.s002.pdf]
