# Supplementary material for: Paternal and maternal psychiatric history and risk of preterm and early term birth: A nationwide study using Swedish registers
Source: PLoS Med. 2023 Jul 20;20(7):e1004256. doi: 10.1371/journal.pmed.1004256 (PMC10358938; doi:10.1371/journal.pmed.1004256)
Supplement: Attachment — Submitted filename: Response_to_reviewers.pdf [file pmed.1004256.s004.pdf]

RE: Ms. No. PMEDICINE-D-22-03876R1

Dear Dr. Philippa Dodd,

Thank you for the opportunity to revise our manuscript entitled “Paternal and maternal psychiatric history and risk of preterm and early term birth: a nationwide register-based study”. We appreciate the constructive comments from the reviewers and the editors. We addressed the comments point-by-point below and accordingly made changes in the manuscript.

We hope that you will find this revised version acceptable for publication.

All authors have contributed to this revision and approved the final version of the manuscript.

On behalf of all authors,

Weiyao Yin MD, PhD

Department of medical epidemiology and biostatistics

Karolinska Institutet, Box 218

171 77 Stockholm, Sweden

Tel: +46-736871443

Fax: +468336981

Email: weiyao.yin.2@ki.se

Requests from the editors:

GENERAL

1. Please respond to all editor and reviewer request detailed below, in full.

Please number the lines in continuous sequence starting at 1 at the top of page 3

Please check carefully throughout for minor grammatical errors "was" instead of "were", for example

Reply:

We appreciate all the insightful comments from the editors and the reviewers which greatly improve our manuscript. We have now addressed all the comments in the revised manuscript, we have reformatted the line number and checked the grammar errors (e.g., the use of plural).

2. Please ensure that the study is reported according to the STROBE guideline and include the completed STROBE checklist as Supporting Information. Please add the following statement, or similar, to the Methods: "This study is reported as per the Strengthening the Reporting of Observational Studies in Epidemiology (STROBE) guideline (S1 Checklist)." When completing the checklist, please use section and paragraph numbers, rather than page or line numbers, as these often change in the event of publication.

Reply:

We did report the study according the STROBE guideline and have now uploaded a STROBE checklist as Supporting Information S1 checklist. The statement is now included (Page 11, line 183, Method).

THE ACADEMIC EDITOR

3. It seems clear to me that this is an interesting paper and that there are 3 positive reviews. Definitely good to send manuscript back to authors with a Major Revision decision. My only additional comment might be to ask the authors to speak a little bit more about mechanism. How might father+mother psychiatric history increase risk over that of just one of them (for example). Authors speak a bit about gene-environment interaction as a mechanism, but it is brief and not entirely convincing. Without a careful consideration of mechanism, paper runs the risk of being a little descriptive.

Reply:

We have now expanded the discussion around mechanisms. We hope this version could help the readers to better understand potential pathways.

Page 15, line 296, Discussion

"…The underlying pathophysiology behind the observed association is complex. The risk of preterm birth is more pronounced in spontaneous than in non-spontaneous delivery. Stress and inflammation/infection are main causes of spontaneous preterm and interact with genetic susceptibility [40]. Maternal stress is also regarded as an important link between maternal psychiatric disorders and preterm birth through endocrine, behavioral, and/or

inflammation/immune mediated mechanisms [5]. Suffering from a psychiatric condition, or having a partner with a psychiatric condition during pregnancy, may be challenging to the mother. Increased psychological stress has been linked to higher levels of stress hormones as cortisol which may induce premature uterus contractions [40, 41]. Accordingly, the highest point estimates for risk of preterm birth is observed for paternal and maternal stress-related disorders. In addition, two meta-analyses show that paternal depression is associated with maternal depression [42, 43]. Studies have identified factors such as violence, substance abuse and smoking as important mediators for poor obstetric outcomes, including preterm delivery, in women with severe mental illness [44-46]. It is possible that the same social and environmental disadvantages that predispose to preterm birth cluster in couples in which both have mental illness [47]. A recent meta-analysis demonstrates that unemployment and financial strain are important risk factors for paternal depression [43]. In line with this, we found that adjustment for parental education, income and age at delivery decreased risks associated with paternal psychiatric history but risk estimates remained essentially unchanged for maternal psychiatric history. This may indicate the involvement of environmental/social factors in the pathway between parental psychiatric history and preterm births, particularly for paternal associations. Still, genetic causes are the main drivers of risk for psychiatric disorders and psychiatric disorders are among the most heritable diseases [6]. There is evidence for genetic components and gene-environment interactions in spontaneous preterm birth [1, 7], and is it not clear if these are associated also with psychiatric conditions. It is possible that, through shared genetic or environmental factors, parents who carry genetic risk for psychiatric disorders may also carry a higher liability for preterm delivery. In our study, the risk of preterm birth increased with number of different psychiatric disorders in a parent. This dose-response relationship may also indicate that higher disease load, either genetic load or as a measure of disease severity, is linked to magnitude of the risk of preterm delivery. Consequently, if genetic, the pattern with lower association in fathers than in mothers suggests a risk driven by interactions between genetic susceptibility and environmental exposures; the genetic risk may in turn be modified by socioeconomic risks and other gene-environmental interactions [7, 8].“

4. DATA AVAILABILITY STATEMENT

   PLOS Medicine requires that the de-identified data underlying the specific results in a published article be made available, without restrictions on access, in a public repository or as Supporting Information at the time of article publication, provided it is legal and ethical to do so.

Reply:

Data source are not freely available because the data was extracted from Swedish national registers, and owing to restrictions by law, data cannot be shared publicly. Data are available from the upon request to the Swedish registers (https://www.socialstyrelsen.se). We have included this statement in the revised manuscript in the Declarations- Availability of data and material.

5. TITLE

Please revise your title according to PLOS Medicine's style. Your title must be nondeclarative and not a question. It should begin with main concept if possible. "Effect of" should be used only if causality can be inferred, i.e., for an RCT. Please place the study design ("A randomized controlled trial," "A retrospective study," "A modelling study," etc.) in the subtitle (ie, after a colon).

Reply:

We have now revised the title as "Paternal and maternal psychiatric history and risk of preterm and early term birth: a nationwide register-based study"

ABSTRACT

Abstract Methods and Findings:

6. Line 14 and 15: are these percentages correct? Please ensure all numerators and denominators used to define percentages are clearly defined for the reader.

Please ensure that all numbers presented in the abstract are present and identical to numbers presented in the main manuscript text.

Reply:

Because the word limit of the abstract, we provided the proportion of preterm births from either parent with a psychiatric history 15,540 (7.05%) in the abstract, but described the details as preterm births from fathers only 4,597 (6.25%), mothers only 8,917 (7.27%) and both parents 2,026 (8.34%) in the main text. We have now provided both numerators and denominators in the abstract to clarify this.

Page 3, line 16, Abstract

"Among 1,488,920 infants, 4,597 of 73,500 (6.3%) were born preterm to fathers with a psychiatric diagnosis, 8,917 of 122,611 (7.3%) to mothers, 2,026 of 24,302 (8.3%) to both parents, and 73,094 of 1,268,507 (5.8%) to when neither parent had a psychiatric diagnosis".

7. Line 15: "…to undiagnosed parents…" as written this could suggest a missed diagnosis which we don't think you mean. Suggest, "…parents unaffected by psychiatric illness." or something similar

Reply:

Thank you for pointing out that this phrase is ambiguous. We have made changes into "…to when neither parent had a psychiatric diagnosis…" in the revised version (Page 3, line 18, Abstract).

8. Line 18: suggest "…were estimated…"

PLOS Medicine requests that outcomes are quantified with p values as well as 95% CIs. When reporting p values please report as p<0.001 or where higher as p=0.002, for example. Suggest reporting statistical information as follows, RR 1.12 (95%CI [1.08, 1.15] p<0. or p=)

Note the removal of the = symbol, the use of parentheses and commas instead of hyphens (as these can be confused with reporting of negative values) to separate upper and lower bounds. Please include any important dependent variables that are adjusted for in the analyses.
In the last sentence of the Abstract Methods and Findings section, please describe the main limitation(s) of the study's methodology

Reply:

Thank you. We have now reported p values for the results and made changes in the statistical information as suggested. The main limitations of the study is mentioned in the Abstract.
Page 4, line 31, Methods and Findings section:
"Despite the large sample size, statistical precision was limited in subgroups, mainly where both parents had specific psychiatric sub-types. Pathophysiology and genetics underlying different psychiatric diagnoses can be heterogeneous. "

Abstract Conclusions:

9. Line 7: suggest "The risk increases when fathers had a positive psychiatric history…" instead

Reply:

Thanks. We made changes as suggested.
Page 4, line 36, Abstract
"The risk consistently increased when fathers had a positive history of different psychiatric disorders, increased further when mothers were diagnosed and was highest when both parents were diagnosed. "

AUTHOR SUMMARY

10. At this stage, we ask that you include a short, non-technical Author Summary of your research to make findings accessible to a wide audience that includes both scientists and non-scientists. The Author Summary should immediately follow the Abstract in your revised manuscript. This text is subject to editorial change and should be distinct from the scientific abstract. Please see our author guidelines for more information.

The author summary should include 2-3 single sentence bullet points under each question.

Reply:

Author summary has now shown in the manuscript following the Abstract.

Author Summary

Why Was This Study Done?

Women with psychiatric diagnoses are at increased risk of preterm birth. It is already known that premature birth is associated with negative health consequences for the offspring.
Less is known about the risk of preterm birth in offspring of fathers with psychiatric diagnoses and for couples where both parents had psychiatric diagnoses. Earlier studies have not thoroughly examined the full range of psychiatric disorders and gestational age.

What Did the Researchers Do and Find?

In a cohort of 1.5 million births, we observed a shift towards earlier gestational age in offspring of parents with a history with psychiatric disorders, particularly for preterm and early-term births.
The risk of preterm birth consistently increased when fathers were diagnosed of different psychiatric disorders (average relative risk=1.12), increased further when mothers were diagnosed (average relative risk=1.31), and was highest when both parents were diagnosed (average relative risk=1.52).
For both the father and the mother, the risk increased in parents diagnosed with several different psychiatric disorders.
The increased risk was present not only for children born preterm, but also for the larger group of offspring born at early term (37-38 weeks) who represent approximately 20% of all births.
What Do These Findings Mean?
Psychiatric diagnoses in both parents are associated with gestational age.
Whether additional social support and prenatal care to the families with a positive psychiatric history could have impact on gestational age requires further investigation.

INTRODUCTION

11. Line 1, page 5: please replace "Background with "Introduction"

Reply: We have made changes as suggested.

12. Please address past research and explain the need for and potential importance of your study. Indicate whether your study is novel and how you determined that. If there has been a systematic review of the evidence related to your study (or you have conducted one), please refer to and reference that review and indicate whether it supports the need for your study.

Reply:
We have mentioned the knowledge gaps and limitations in previous studies to motivate the need for our study in the Introduction Paragraph 1. In the revised manuscript we have referred to a recent systematic review in the introduction (Page 6, line 66,) : "One recent systematical review correlated mental illness before delivery with pregnancy complications including preterm delivery, with most evidence from depression and anxiety disorders [8]. "

METHODS and RESULTS

13. Page 6, line 21: "…ultrasound examination is routinely offered," would it be worth stating that this is to determine gestational age, for clarity.

Reply:
We made changes into "Since 1990, early second-trimester ultrasound examination ultrasound examination is routinely offered to determine gestational age…", as suggested (Page 8, line 113).

14. Page 9, line 1: please remove the semi-colon after the sub-heading
Reply: Semi-colon removed as suggested.

15. Lines 4-7: as above, percentages presented don't seem to correlate with the numerators and denominators presented here. Please clearly define the numerators and denominators used to derive percentages. Apologies if I have misunderstood something but perhaps all the more reason to revise/clarify!
Reply:
We apologize for any ambiguity or confusion about the percentages reported here. The results are correspondent to the numbers and percentages in the Table 1. We have now reported the numerators together with the denominators.
Page 11, line 189, Results
"At birth, 4,597 of 73,500 (6.3%) offspring were born preterm to fathers with a psychiatric diagnosis, 8,917 of 122,611 (7.3%) to mothers with a psychiatric diagnosis, 2,026 of 24,302 (8.3%) to both parents, and 73,094 of 1,268,507 (5.8%) to neither parent with a psychiatric diagnosis."

16. As above, PLOS Medicine requests that outcomes are quantified with p values as well as 95% CIs, including in tables and figures (please see below). When reporting p values please report as p<0.001 or where higher as p=0.002, for example.
Reply:
Thank you. We have now reported all the risks with p-values and 95% confidence intervals.

TABLES
Please ensure that each figure caption affiliated to tables clearly describes their content without the need to refer to the text.
where 95% CIs are reported please also report p values. Please do not use asterisks to represent p values. Please report p as p<0.001 or where higher as p=0.002, for example. If not reporting p values, for the purpose of transparent data reporting, please clearly state the reasons why not.
Table 1: to improve accessibility to the reader, suggest removing the % symbols, except in column headers or row headers where you define (%) for the reader.
Please see reviewer #1 comments regarding reporting of income which we agree with
Maternal/paternal age at delivery please include (years) presumably?
Table 2: As above, please include p values with 95% CIs. Please ensure that upper and lower bounds of CIs are reported on the same row to improve reader accessibility.
We suggest separating upper and lower bounds by commas instead of hyphens as these can be confused with reporting of negative values.
Reply:
As required, we have now added p values to all the tables and results, removed the % in the Table 1, used commas instead of hyphens for confidence intervals, to our best ability adjusted

the CIs on the same row, and defined the unit of maternal/paternal age at delivery (years) as well as income (yearly income, SEK).

FIGURES

Please ensure that each figure caption clearly describes the figure content for the reader without the need to refer to the text.

Figure 1: in the figure caption please indicate that your analyses are unadjusted. Please consider avoiding the use of green and/or red to make the figure more accessible to those with colour blindness.

Figure 2 and 3: in the figure caption please clearly define the meaning of the dots (RR) and lines (95% CI) for the reader. Suggest that the size of the lines Vs the dots be more proportionally presented to look more like lines rather than blocks

Reply: The Figure 1 has now been moved to S1 Appendix as Figure B. In the revised version, the Figure 1 shows the adjusted quantile regression plots. The information has been described in the figure legends (Page 26, Figure 1). Other comments have been addressed as required (e.g., colors, figure captions and lines).

DISCUSSION

Line 17, page 10: "In this prospective nationwide birth cohort…" this is not a prospective study. Please revise. Suggest, "…observational cohort study…" or something similar

Line 9, page 11: "for the first time" please avoid assertions of primacy which can be risky. Suggest to the best of our knowledge, or similar.

Line 12, page 12: please remove the sub-heading "Conclusion"

Please remove details of funding, conflict of interest and data availability statements from the end of the discussion and include only in the manuscript submission form when you re-submit your manuscript.

Please remove the patients and public involvement and transparency statements all together.

Please move your ethics statement to the methods section of the main manuscript.

Reply:

"In this prospective nationwide birth cohort…"

Thank you for raising the concern about the definition of the study design. The data was collected in the clinics across Sweden, ***prospectively***, as it happened. The prospective nature of the data collection was reflected by our analyses. Therefore, we used "prospective" to emphasis that no recall bias in the recording of gestational age or exposures. In the revised version, we have moderated the language by rephrasing from "prospective cohort" to "population-based cohort", or as in the title "nationwide register-based study".

"for the first time"

Thanks for raising the concern for the possible assertions. We have now changed the sentence to "…and to examine, to the best of our knowledge, the additive effect of maternal and paternal exposure for the first time…" (Page 14, line 261, Discussion)

Remove the “Conclusion”, funding, conflict of interest, data availability statement and other declarations.
We removed these statements accordingly.

REFERENCES
For in-text reference callouts citations should be placed in square brackets preceding punctuation, as follows “for example [1,3,9].” Please note the absence of spaces between citations.
In the bibliography, please ensure that up to but no more than 6 author names are listed followed by et al., in the event that more than 6 authors contribute to an individual study.
Please see our website for other reference guidelines
Reply: We have updated the references as required.

We thank the academic editor for his/her positive feedback that improved our manuscript.

Reviewer #1: Alex McConnachie
Yin et al report on a large retrospective study of the association between a history of psychiatric diagnoses and preterm birth, using nationwide data from Sweden. This review considers the statistical aspects of the paper.

Generally, these are very good. The data sources and statistical methods are well described, and the results are presented quite clearly. My comments are fairly minor.
Reply: We thank the positive comments from the reviewer.

1. Conceptually, I would see this more as a case-control study, than a cohort study, since the population is defined by the outcome, rather than the initial condition. This is a study of all live births, not a study of all pregnancies. Equally, I would describe this as a retrospective study, not prospective, since the study is being conducted after the data have been collected. A prospective cohort study would have identified all pregnancies as they happened, and followed them up to outcome. Quite rightly, the authors note the potential bias from not knowing about miscarriages.

Reply:
Thank you for raising the concern about the definition of the study design. The study population includes all live-born births to Nordic parents from 1997 to 2016 in Sweden. The population is not defined by the outcome neither by the exposure. It is true that the data is available when the study starts. However, the data was collected in the clinics across Sweden, ***prospectively***, as it happened. Furthermore, our analysis reflects the sampling and does not condition on any future events, e.g., by conditioning on the outcome as in a case-control study. It is true that we only have live births in our database. Even though this may introduce a selection bias with consequences we can only speculate about, it does not change the prospective nature of the data collection and analysis procedure.

In the revised version, we have now moderated the language by rephrasing from "prospective cohort" to "population-based cohort", or as in the title "nationwide register-based study".

2. At times, the authors stray into using causal language, such as in the conclusion that "Psychiatric diagnoses in both parents impact gestational age", which should be avoided.

Reply:
Thank you for reminding us. We agree that the causal language should be avoided. The sentence has been deleted in the revised version. The current conclusion: "Paternal and maternal psychiatric history were associated with a shift to earlier gestational age and increased risk of births before full term…" (Page 4, line 35, Conclusion under Abstract).

3. I note that the exposure includes psychiatric diagnoses during pregnancy. Would there be any value in looking only at long-standing diagnoses as a sensitivity analysis? Or just at episodes during the pregnancy?

Reply:

Thank you for the constructive suggestion. To address this concern, we have performed two sensitivity analyses. We restricted the analysis to incident parental psychiatric disorders diagnosed before two years of conception. Next, we removed diagnosis that only happened once, or only diagnosed during a short time period, e.g., one month, to further clarify the robustness of the association. We have included this analysis in the revised version (Table J and Table K in S1 Appendix).

Page 10, line 174, Method

"(6) To examine the specificity of timing of the diagnosis, we restricted the analysis to parental psychiatric disorders first diagnosed before two years of conception. (7) To see how much the results were driven by individuals with provisional diagnoses, i.e, only one single diagnosis in a individual or diagnoses given over a short period of time, we repeated the main analysis but excluded parental psychiatric diagnoses recorded only once in the patient register or some diagnoses within 30 days."

Page 13, line 237, Results

"When restricting to singleton births (Table I in S1 Appendix), to psychiatric diagnoses first being diagnosed two years before conception (Table J in S1 Appendix), to diagnoses occurred at least twice lasting over 30 days (Table K in S1 Appendix) and to births from 2005 onwards (Table L in S1 Appendix), the estimated preterm risks were essentially similar to that in the main analysis."

4. The statistical analysis section begins by talking about looking at the shift in distribution of gestational age at delivery, which is good, but this is only done graphically. No regression is attempted that looks at GA as a continuous outcome. The authors do go beyond a binary logistic regression to look at GA in 4 categories, but they analyse this with a multinomial regression model. Would it make more sense to use an ordinal regression approach? Would this get closer to looking at the shift in distribution? Saying that, the current categorisation only separates out different degrees of preterm birth - three quarters of the population remain in a single group, so an ordinal regression is not looking at a shift across the whole distribution, only a shift into the left-hand tail.

Reply:

You raise an important point. To address this fully we have fitted quantile regression models to the GA data distributions. The quantile regression allows an examination of effects of exposures at each quantile, especially the lower/higher quantiles; in our study, lower GA. Using the results from the quantile regression, we plotted the shift of GA in days, comparing offspring GA of parents with psychiatric diagnosis to that of parents without psychiatric

diagnosis, adjusted for offspring's birth year (categorized in 5-year intervals). We have now made changes accordingly in the Method, Results and Discussion.
Page 9, line 130, Method
"To address potential changes in the full gestational age distribution, without dichotomizing the data, we show the shape of the gestational age distribution for the different exposure groups by calculating probability density functions. A density curve provides a visualization of what percentage of observations in the gestational age distribution fall between different values (here: gestational weeks). Next, we fitted quantile regression models adjusting for offspring's birth year (categorized in 5-year intervals) to examine the effects of exposures at different quantiles. Where a linear regression is analysing differences in the mean between exposures, quantile regression can compare exposures at an arbitrary cut-off of a data distribution, e.g at the 1st, 5th or 10th percentiles, and study effects on the most sensitive lower tail of the gestational age distribution. Thus, for the analysis of gestational age, the quantile regression can estimate at which gestational age (week) the 1%, 5% or 10% earliest born children was delivered and how this age differ between exposures.. "

Page 11, line 202, Results,
"Overall, compared to offspring of neither parent with a psychiatric diagnosis, the gestational age distribution was shifted towards earlier gestational age for offspring of parents with psychiatric history, particularly for a psychiatric history in mothers and in both parents and for the lower quantiles of gestational age, e.g., quantiles lower than 10%tile, approximately 38 weeks of gestational age (Figure 1 and Figure B in S1 Appendix)."

Page 13, line 244, Discussion
"…we observed a shift towards earlier gestational age in offspring of parents with a history of psychiatric diagnosis, particularly at lower quantiles of gestational age corresponding to preterm and early term births."

5. The authors choose to report unadjusted associations as primary results, with adjusted associations as secondary, but they do not make much of these secondary results. I have not reviewed every result in detail, but my impression is that the maternal associations are relatively unaffected by these adjustments, whilst the paternal associations, even if they remain statistically significant, are often noticeably reduced after adjustment.

Reply: Thank you for raising this concern. We considered covariates such as parental education, income and age at delivery as mediators in the pathway from parental psychiatric disorders before delivery to preterm births. Therefore, we reported risk estimates from Model 1 where birth year was adjusted for, as our primary results. Thank you for pointing out that after adjustment, paternal associations were reduced to a greater extent than maternal associations. It may indicate the role of adjusted covariates in the pathway of paternal associations. We have now described this in the results and also a brief discussion.

Page 12, line 210, Results
"…, Additionally, adjusting for maternal and paternal age, income and education, the RR was similar for maternal associations 1.27 (1.24-1.29] p<0.001), but noticeably reduced for paternal associations RR=1.04 (1.01-1.07] p=0.006) (Table 2). Adjusting further for maternal BMI and maternal smoking did not further alter the results (Table C in S1 Appendix)."

Page 16, line 311, Discussion
"In line with this, we found that adjustment for parental education, income and age at delivery decreased risks associated with paternal psychiatric history but risk estimates remained essentially unchanged for maternal psychiatric history. This may indicate the involvement of environmental/social factors in the pathway between parental psychiatric history and preterm births, particularly for paternal associations. "

6. In the results, it states that "4,597 (6.25%) of those born preterm had fathers with psychiatric history". Is this the wrong way round? I.e. 6.25% of those who had fathers with psychiatric history were born preterm? The same thing applies to the rest of that sentence.

Reply: We apologize for any lack of clarity and confusion about the percentages reported here. The results are correspondent to the numbers and percentages in the Table 1. We have now reported the numerators together with the denominators.
Page 11, line 189, Results
"At birth, 4,597 of 73,500 (6.3%) offspring were born preterm to fathers with a psychiatric diagnosis, 8,917 of 122,611 (7.3%) to mothers with a psychiatric diagnosis, 2,026 of 24,302 (8.3%) to both parents, and 73,094 of 1,268,507 (5.8%) to neither parent with a psychiatric diagnosis."

7. The modelling treats parental psychiatric history as a 4-level categorical variable (neither, mother only, father only, both) but would it make sense to look at maternal and paternal psychiatric histories as independent predictors? Is there any evidence that the combination of both parents having a psychiatric history is any more (or less) than the sum of the two separate associations? The authors use "dose-response" to talk about the associations across the four categories used, but I do not think that is quite the correct term, since the exposure is not numeric. It makes more sense when talking of associations with the number of maternal and paternal diagnoses. It could work if describing the association with the number of parents (0, 1, or 2) with a diagnosis.

Reply:
4-level categorical variable
There may be positive or negative interaction between maternal and paternal exposure, on an additive scale or a multiplicative scale (*Knol MJ, VanderWeele TJ. "Recommendations for presenting analyses of effect modification and interaction". 2012*). Therefore, the sum of two separate associations are not always equal to the joint association. Using 4-level categories, we completely separated maternal (or paternal) exposure from paternal (or maternal) exposure

to study how the risks differs and adds up between the couples. We haven't formally tested the additive or multiplicative interactions in the present study considering that we have already a lot of materials for the readers. We would like to add this analysis if the reviewer and the editor think it necessary.

"dose-response"

Thank you for correcting us. We agree that "dose-response" relationship is not a suitable term to describe exposure in fathers, mothers and both parents. In the revised manuscript, we have avoided the term "dose-response" when describing the risk pattern for father only, mother only and both, but only keep it when reporting the results for co-occurring different psychiatric disorders in a parent.

8. The discussion section starts by saying that there was a shift towards earlier gestational age associated with parental history of psychiatric diagnoses. I am not sure this is a fair interpretation. The regression analyses only look at degrees of preterm birth, not the whole distribution, so all you can say is that parental history of psychiatric diagnoses is associated with an increased risk of preterm birth. This could be due to a shift in the distribution, but if parental psychiatric history was associated with an increase in the variance of gestational age at birth, but not the mean, then the same pattern could result. Regression analyses of gestation age at birth as a continuous variable would let you say there was a shift, but that might add a lot of material to the paper.

Reply: Thank for your suggestions. We agree and have added analysis using quantile regressions. Please see the reply to Comment 4.

9. Table 1 reports income to 6 significant figures. Is this too much? Also, what are the units?

Reply: The income is defined as yearly income. The unit is Swedish Krona (SEK). We have now clarified this in the Method (Page 8, line 125, Other covariates) and in Table 1.

10. In the contents for the supplement, the title of eTable 2 is wrong. In eTable 2 itself, why only report the lower percentiles? Why not show 50th, 75th, 90th, and 95th as well? Would that give a more complete description of the distribution, and the shift associated with parental psychiatric history?

Reply: We apologize for the negligence. We have now corrected the title. In previous version, we reported the lower percentiles because we consider the shift in earlier gestational age (GA) of most research interest, and percentile 25% is approximately correspondent to GA of 39 weeks. As suggested, we have now included the 50th, 75th, 90th and 95th in the new Table (Table B in S1 Appendix). In addition, we have also performed a quantile regression to address this issue (See Comment 4, Figure 1).

We thank Dr. Alex McConnachie for his/her constructive comments that improved our manuscript.

Review #2
This study investigates an understudied but important area, the contribution of paternal factors to pregnancy outcomes and offspring health. It has the advantage of large numbers by using the National Patient Register in Sweden. Psychiatric health is a timely topic given the reported increase in disorders during the pandemic.
Reply: We thank for the reviewer's positive comment.

1. In the introduction the paper highlights a data gap as "the association between maternal psychiatric history and preterm delivery has not been extensively studied across a wide range of psychiatric disorders. Rather, previous studies have generally focused on mood and anxiety disorders." This statement is not entirely accurate, as reference 4 by Männistö et al found an increased birth associated with maternal bipolar disease and schizophrenia. The present analysis does expand on additional disorders, although I would defer to a psychiatrist about the categories. I think the main novel contribution of the current paper is the ability to study paternal psychiatric disorders.

Reply:
Thank you for the comment. The study of Männistö et al is indeed the one most comparable to us. They have studied several subgroups of psychiatric disorders including depression, anxiety, bipolar, schizophrenia and unspecific diseases. We have rephrased the sentence and included Männistö's publication as one of the studies that have examined other psychiatric disorders than depression and anxiety.
Page 6, line 66, Introduction
"One recent systematical review correlated mental illness before delivery with pregnancy complications including preterm delivery, with most evidence from depression and anxiety disorders [8]. Rather, most previous studies have generally focused on mood and anxiety disorders [8-14], with other psychiatric disorders receiving less attention [4, 15-22]."

Defer to a psychiatrist about the categories
We have referred to psychiatrists who are co-authors of the study, and changed the category of "schizophrenia and other paranoid psychoses" into "schizophrenia and other non-mood psychotic disorder". We also re-defined "neurodevelopmental disorders" as "neurodevelopmental disorders, emotional and behavioral disorders of childhood origin and intellectual disability" (Page 8, line 107, Method; Table A in S1 Appendix). Thanks for your advice.

Paternal psychiatric disorders
We agree that paternal associations is the main novel contribution of our study and now described this point as the second knowledge gap.

2. The same comment about the 2nd data gap in the introduction. The current paper uses the same plots as the Männistö paper (ref 4), so it's not truly a data gap. I think the introduction would benefit from highlighting the 3rd data gap which is the most novel.

Reply:

Thanks for the comment. As we know, the study of Männistö et al is the only one who also examines the risk of births before full term, but they did not cover the full psychiatric categories and only for mothers with psychiatric disorders. We used density plots to show changes in distributions of the mean gestational age as they did. In the revised manuscript, we performed quantile regressions to examine the shift of distribution of gestational age at lower quantiles, which corresponds to earlier weeks of gestational age. We have now adjusted the description for this knowledge gap accordingly (Now presented as the 3rd data gap).

Page 6, line 75, Introduction

"Third, evidence suggests that advancing birth, even by as little as 1 or 2 weeks before full term, can affect neonatal morbidity [23] and future health outcomes [2]. How the risk of preterm delivery in offspring of parents with psychiatric diagnoses extends to the entire range of gestational age is largely unknown, particularly at early term (37-38 weeks). We are only aware of one study that has examined the risk of birth before full term for several psychiatric disorders in mothers [4]. No studies have examined changes in distribution of gestational age for paternal psychiatric disorders, and shift of gestational age in preterm and early-term births. Support for such an association will identify a large group at risk with unmet needs. "

Methods

3. The definition of spontaneous and non-spontaneous preterm birth needs to be added to the "Psychiatric diagnoses and preterm birth" section on p. 6.

Reply:

We briefly described the definition in the section of Method, other covariates in the old manuscript. As suggested, we have now added details and included it under the section Method, Psychiatric diagnoses and preterm birth.

Page 8, line 118

"We classified deliveries as spontaneous and non-spontaneous, i.e. spontaneous initiation of labor, or through induction of labor or cesarean section, according to the record of onset of labor in the MBR."

4. It's not clear to me why cubic splines were used for birth year with 5 degrees of freedom. Perhaps it would help to add a reference. I defer to a statistician.

   Reply:

   We used the natural cubic splines to model continuous variables with 5 degrees of freedom due to a concern with residual confounding potentially introduced when categorizing measures on a continuous scale. The 5 degree of freedom was chosen as a default. We referred to the method from Benedetti A and Abrahamowicz M which was

cited in the section of Method, Statistical analyses (Page 9, line 144), as written already in the analysis plan before starting analysis (S1 text).

Results

5. All of the covariates included in the models should be provided in Table 1 - for example, BMI, smoking, etc. Also, preterm birth is an outcome and outcomes are usually presented in a separate table, not Table 1.

Reply:

We have now included information for maternal BMI at the first prenatal visit, maternal smoking during pregnancy, spontaneous delivery and multiple births in the Table 1. We described accordingly in the text.

Page 11, line 199, Result

"Their mothers were more likely to be underweight or obese, to have smoked during pregnancy, and to have spontaneous labor (Table 1)."

We included preterm birth and gestational age in the Table 1 to provide a summary of descriptive information of the study population for convenience of the readers. For example, for simplicity, the relative risks for different gestational age categories were shown as forest plot in Figure 3, without numbers of total subjects, outcomes and percentages; but these information can be found in Table 1. However, we are happy to remove gestational age in the Table 1 if the reviewer and the editor think it is more appropriate.

6. This point is minor but only 1 decimal place is needed for percents, particularly the % preterm birth (p. 9. Lines 5-7).

Reply: As suggested, we reported only 1 decimal in the percentage in the revised text.

7. Suggest using an alternative terminology for the reference group instead of "Undiagnosed parents". Perhaps "Neither parent with psychiatric disorder" or something along those lines. Also, is it "psychiatric history" or current "psychiatric disorder"? "History" means something different. Need to be technically precise with the terminology.

Reply:

Thank you the comment. We used psychiatry history defined as any psychiatric diagnosis before the birth of the child. We want to emphasis the nature of 'history' as an exposure to a psychiatric diagnosis before delivery. In the revised manuscript, we have changed the term "Undiagnosed parents" to "neither parent with a psychiatric diagnosis" or "when neither parent had a psychiatric diagnosis" when describing the reference group.

We thank the Reviewer 2 for his/her constructive comments that improved our manuscript.

Reviewer #3
This study examines associations between parental psychiatric diagnoses and length of gestation. The study makes a valuable contribution to the field, and the results eloquently illustrate dose-response relationships between psychiatric diagnoses of the parents individually and in combination in relation to shortened gestation. Most of my comments are focused on language for presenting the methods and findings.
Reply: We thank the reviewer for the positive comments.

1. In the main descriptive results in the abstract and manuscript, the denominators for the percentages are not clear. In addition, it is not immediately clear where the numerator 15,540 in the abstract comes from. Accordingly, please explicitly specify that 15,540 infants were born preterm from parents one or both of whom had a psychiatric history. In addition, please ensure that it is clear that the percentages in the sentences beginning at line 14 in the abstract (page 3) and line 5 of the results (page 9) refer to the PTB rates within exposure strata, not the size of the exposure strata as a proportion of the overall analytic sample. I recommend reporting the total number of preterm infants and the overall PTB rate (at least in the main manuscript) for context. Finally, to improve clarity I would change "No parents" to "Neither parent" in the column heading in Table 1.

Reply:
We apologize for the lack of clarity in the previous version. We have now reported numerators and denominators and specify the parental exposure group. We changed ”no parents” to ”neither parent” as suggested.
Page 3, line 16, Abstract
”Among 1,488,920 infants, 4,597 of 73,500 (6.3%) were born preterm to fathers with a psychiatric diagnosis, 8,917 of 122,611 (7.3%) to mothers, 2,026 of 24,302 (8.3%) to both parents, and 73,094 of 1,268,507 (5.8%) to when neither parent had a psychiatric diagnosis”.
Page 11, line 189, Results
"At birth, 4,597 of 73,500 (6.3%) offspring were born preterm to fathers with a psychiatric diagnosis, 8,917 of 122,611 (7.3%) to mothers with a psychiatric diagnosis, 2,026 of 24,302 (8.3%) to both parents, and 73,094 of 1,268,507 (5.8%) to neither parent with a psychiatric diagnosis."

2. Given the heterogeneity in the literature around different GA windows and designations of PTB severity, I would explicitly define early term birth in the abstract (as you do at line 14 of page 5).

Reply: Thank you. We have now defined the early term in the abstract.

3. Please standardize the formatting of reference numbers to ensure consistency (some but not all are currently in superscript).

Reply:

We have now standardized the format according to the requirements of the journal in the revised version.

4. Lines 5-6: "Paternal support during pregnancy has been associated..."

Reply:
Thank you. We have adjusted the sentences to ”Paternal involvement during pregnancy has been shown to moderate maternal stress, and the lack of such paternal support is associated with adverse perinatal outcomes” (Page 16, line 328, Discussion)

5. Page 7, line 11: gestational age densities - this terminology is unclear to me, and I'm not sure whether it's a standard term. Perhaps "gestational age distributions"?

Reply:
In the revised version, besides density curve, we have added quantile regressions to capture changes in the gestational age distributions for different exposure groups. Therefore, we have also adjusted the text describing the method. The term "gestational age densities" has been changed to "gestational age distributions".
Page 9, line 130, Method
”To address potential changes in the full gestational age distribution, without dichotomizing the data, we show the shape of the gestational age distribution for the different exposure groups by calculating probability density functions. A density curve provides a visualization of what percentage of observations in the gestational age distribution fall between different values (here: gestational weeks).”
Figure B in S1 Appendix, Page 19, Title
”Density curve of gestational age (GA) distributions by parental psychiatric history”

6. Page 5, lines 9-10: relied on modest sample sizes with mixed results - I recommend adjusting this to "relied on modest sample sizes and yielded mixed results"

Reply: We have changed accordingly (Page 6, line 70). Thank you.

7. In the methods (abstract and main manuscript), please clarify the definition of "Nordic parents", as it's unclear whether this refers to ethnicity, residence, citizenship, or another definition. If this inclusion criterion is based on ethnicity, that would need to be justified.

Reply:
Thank you for making us aware of this lack of clarity. The inclusion criteria is not based on ethnicity. We have now clarified this in the Method.
Page 7, line 89, Method,
”We included all infants born alive in Sweden from 1997 to 2016, to Nordic parents (parents born in Sweden, Finland, Norway, Denmark and Iceland),… ”

8. Please describe the follow-up time during which exposures (psychiatric diagnoses) were measured, and whether this varied within the study population. Given that outpatient

diagnoses were available beginning in approximately 2001, births at the end of the study period would presumably have more complete information on outpatient psychiatric diagnoses than those at the beginning of the study period. It would likely also be informative to report how long before delivery the most recent diagnosis tended to be in mothers and fathers who had a prenatal psychiatric diagnosis (e.g., knowing how often you had to look back further than 2 years to find a diagnosis would give a measure of how many diagnoses would be missed in patients with <2 years of prenatal data).

Reply:

We have added a few complementary analyses to address your questions. To illustrate how the association between psychiatric diagnoses and gestational age may change over time, we have now repeated the analyses for 'any psychiatric diagnosis', limiting the follow-up to births from 2005 onwards (Table L in S1 Appendix). To better understand how the diagnoses are distributed in relation to the dates of delivery, we report the cumulative distribution of years between the birth and last previous diagnosis for mothers and fathers separately (Figure C in S1 Appendix). In the revised version, we added information about the year since the first and the last diagnosis in relation to the date of delivery.

Page 10, line 179, Method,
"We restricted the population to births from 2005 onwards"

Page 11, line 192, Results,
"The median lag years between the first observed diagnosis and the date of delivery was 7 years (25th to 75th percentiles: 4 to 13 years) in fathers and 6 years (25th to 75th percentiles: 3 to 11 years) in mothers; the corresponding years for the last observed diagnosis was 2 year (25th to 75th percentiles: 0.6 to 6 years) years in fathers and 2 year (25th to 75th percentiles: 0.6 to 5 years) in mothers. The cumulative distribution of years between birth of the child and last previous diagnosis for mothers and fathers separately was shown in Figure C in S1 Appendix."

Page 13, line 237, Results,
"When restricting to singleton births (Table I in S1 Appendix), to psychiatric diagnoses first being diagnosed two years before conception (Table J in S1 Appendix), to diagnoses occurred at least twice lasting more than 30 days (Table K in S1 Appendix) and to births after 2005 (Table L in S1 Appendix), the estimated preterm risks were essentially similar to that in the main analysis"

The Swedish health system applies universal access to care that is allocated according to need. All visits to a specialist are recorded in the patient register and it is these data that we are using in our analyses. The patient registry is therefore an excellent source for analyzing incident cases of various medical conditions, including psychiatric ones. However, it can be

more difficult to interpret these diagnoses to understand severity and how the patient is feeling at any given time. For example, many, but not all, patients come in for regular appointments while others come in as emergencies. After assessment by a specialist, many individuals do not need specialist care at any given time but can be served by general practice and these we do not see at all. However, what is important for our analyses is that we trust that they are correct. The fact that we miss some individuals' diagnoses and perhaps even miss a number of individuals entirely, we do not see as a major problem for answering the purpose of our study. Possibly these individuals can introduce measurement errors which could result in, if anything, underestimating the actual associations. It would be more problematic with biases in the other direction. We have mentioned this limitation in the discussion (Page 14, line 266).

Furthermore, to see how much our results are driven by individuals with only a single diagnosis or some diagnoses given over a short period of time, we have repeated the main analyses in Table 2 but in a dataset where we deleted diagnoses that occurred only once in an individual or diagnoses within 30 days (Table K in S1 Appendix).

9. Last 2 sentences of page 6: I recommend rephrasing this to "We categorized gestational age into preterm (<37 weeks) 23 and term (≥37 weeks) birth, and then further into very preterm (<32 weeks), moderate to late preterm (32-36 weeks), early term (37-38 weeks) and full term (≥39 weeks).

Reply: We have made changes in the revised version (Page 8, line 115, Method).

10. Page 7, line 5 - change "was obtained" to "were obtained"

Reply: We have corrected this grammatical error in the revised version (Page 8, line 124, Method).

11. Page 7, line 8 - change to "...≥ 3 years postgraduate education, PhD education) were defined at delivery."

Reply: We have corrected this grammatical error in the revised version (Page 8, line 127, Method).

12. Page 8, line 11 - add "therefore" before "we did not include them..."

Reply: We have made changes in the revised version (Page 9, line 162, Method).

13. The authors report on statistical significance in several places where the point estimates are more important:

Page 9, line 18 - instead of reporting that associations remained statistically significant in adjusted analyses, please describe the changes in point estimates.

Reply:

We have provided the point estimates after adjustment in the revised version.

Page 12, line 210, Results
”Additionally, adjusting for maternal and paternal age, income and education, the RR was similar for maternal associations 1.27 (1.24-1.29] p<0.001), but noticeably reduced for paternal associations RR=1.04 (1.01-1.07] p=0.006) (Table 2).”

14. Similarly, page 10, lines 13-14: The estimated preterm risks remained when restricting to singleton births (eTable 9). Please describe any changes in the point estimates in the restricted model compared to the principal analysis.

Reply: We have updated accordingly.
Page 13, line 237, Result
”When restricting to singleton births (Table I in S1 Appendix), …, the estimated preterm risks were essentially similar to that in the main analysis.”

15. Top of page 11: After adjustment ..., the risk remained statistically significant. Please describe if and how the point estimates changed in the adjusted models rather than focusing on the absence of a change in statistical significance.

Reply:
We updated accordingly. Thank you for pointing out this.
Page 13, line 251, discussion
”After adjustment for parental age and socioeconomic status and maternal BMI and smoking, the risk was largely similar for maternal associations, but noticeably reduced for paternal associations, although remained statistically significant... ”

16. Page 11, line 3: I recommend adding a hyphen in "sex-specific differences"

Reply: We changed accordingly (Page 13, line 255, Discussion).

17. Page 11, sentence at lines 21-22. Please clarify that "both the parents" refers to psychiatric diagnoses for the parents.

Reply: We have now clarified this as ”…by psychiatric diagnoses for both the parents” (Page 14, line 276, Discussion).

18. Page 11, last paragraph: To improve clarity, I recommend setting off the text "in Sweden approximately 20% of all births" between dashes (—) instead of commas.

Reply: We updated accordingly (Page 14, line 278, Discussion).

19. Page 12, lines 6-7: I recommend adjusting the text to read "...suggested paternal depression as a risk factor for preterm birth (24), but not for paternal schizophrenia"

Reply: We adjusted accordingly (Page 15, line 286, Discussion).

Page 12, liners 8-9: Adjust to read "Increased risk of preterm delivery in mothers with a psychiatric history has been reported..."

Reply: We adjusted accordingly (Page 15, line 289, Discussion).

Page 13, line 4: Remove the comma after "Sweden" and add a comma after "psychiatric diagnosis(es)"
Reply: We adjusted accordingly.

Page 13, line 8: Are the results generalizable beyond Sweden? Phrasing this as a question strikes me as inappropriately informal, so I would consider re-phrasing to summarize what we do or don't know about the generalizability of the study findings.
Reply:
Thank you. We have re-phrased the sentences to "Causally, if we speculate that inherited genetic risk as well as 'biological' or 'psychological' stress can carry over to other populations, the results are generalizable beyond Sweden…"(Page 17, line 336, Discussion)

Finally, I recommend rephrasing the second sentence of the conclusion (page 13, lines 14-15), as it seems to read awkwardly beginning at "even higher".
Reply: We have changed the sentence
Page 4, line 36, Abstract Conclusion
From
"The risk increase when fathers had psychiatric history, even higher when mothers were diagnosed and highest when both parents were diagnosed."
To
"The risk consistently increased when fathers had a positive history of different psychiatric disorders, increased further when mothers were diagnosed and was highest when both parents were diagnosed."

We thank the Reviewer 3 for his/her patient editing and positive feedback that improved our manuscript.
