# Supplementary material for: Paternal and maternal psychiatric history and risk of preterm and early term birth: A nationwide study using Swedish registers
Source: PLoS Med. 2023 Jul 20;20(7):e1004256. doi: 10.1371/journal.pmed.1004256 (PMC10358938; doi:10.1371/journal.pmed.1004256)
Supplement: Attachment — Submitted filename: Reply_letter_R2.pdf [file pmed.1004256.s005.pdf]

RE: Ms. No. PMEDICINE-D-22-03876R2

Dear Dr. Philippa Dodd,

Thank you for the opportunity to revise and resubmit a further improved version of our manuscript entitled “Paternal and maternal psychiatric history and risk of preterm and early term birth: a nationwide study using Swedish registers” for publication in the PLOS Medicine.

We have now addressed the additional comments of the editors and reviewers below and accordingly made changes in the manuscript. We hope that you will find this revised version of the manuscript acceptable for publication.

All authors have contributed to this revision and approved the final version of the manuscript.

On behalf of all authors,

Weiyao Yin MD, PhD

Department of medical epidemiology and biostatistics

Karolinska Institutet, Box 218

171 77 Stockholm, Sweden

Tel: +46-736871443

Fax: +468336981

Email: weiyao.yin.2@ki.se

Requests from the editors:

GENERAL

TITLE

1. Please make reference to the study's country of origin in the title, for example.'…a nationwide register-based study from Sweden' or '…a nationwide study using Swedish registers'

Reply:

As suggested, we have now revised the title as 'Paternal and maternal psychiatric history and risk of preterm and early term birth: a nationwide study using Swedish registers'.

ABSTRACT

2. Thank you for your clarifications around numerators, denominators & percentages reported here. We suggest for additional clarity detailing the number of preterm births in those unaffected by psychiatric illness before detailing those that are affected, for example (or similar): 'Among the 1,488,920 infants born throughout the study period, 1,268,507 were born to parents without a psychiatric diagnosis and 73,094 (5.8%) were born preterm. 4,597 of 73,500 (6.3%) infants born to fathers with a psychiatric diagnosis were preterm. 8,917 of 122,611 (7.3%) infants were born preterm to mothers with a psychiatric diagnosis and 2,026 of 24,302 (8.3%) infants born to both parents with a psychiatric diagnosis were born preterm.' Please also amend the same piece of data reporting in the results section of your manuscript (line 188 onwards).

Reply:

We have now reported the numbers of offspring without parental exposure before detailing those with parental exposure. Changes have been made accordingly in the Abstract (line 16) and Results (Line 195).

AUTHOR SUMMARY

3. Thank you for including an author summary. Please include bullet points preceding each individual statement. Line 44 – please make sentencing beginning 'Earlier studies…' a separate bullet point Line 49 – suggest diagnosed 'with' instead of 'of' Line 50 suggest '(average relative risk [RR] =1.12)' and thereafter 'average RR…' please note however, comments from reviewer #2 (please see below) regarding the presentation of average RR Vs the range of risk, which we agree with. Line 52 – sentence beginning 'For both…' suggest making this a separate bullet point. Line 57 – 'Psychiatric diagnoses in both parents are associated with gestational age.

Reply:

We have now revised the Author Summary (line 42).

Author Summary

Why Was This Study Done?

- Women with psychiatric diagnoses are at increased risk of preterm birth. It is already known that preterm birth is associated with negative health consequences for the offspring.
- Less is known about the risk of preterm birth in offspring of fathers with psychiatric diagnoses and for couples where both parents had psychiatric diagnoses.
- Earlier studies have not thoroughly examined the full range of psychiatric disorders and gestational age.

What Did the Researchers Do and Find?

- In a cohort of 1.5 million births, we observed a shift towards earlier gestational age in offspring of parents with a history with psychiatric disorders, particularly for preterm and early-term births.
- The risk of preterm birth consistently increased when fathers were diagnosed with different psychiatric disorders (relative risk (RR) =1.12, 95% confidence interval (CI) [1.08, 1.15]), increased further when mothers were diagnosed (RR =1.31, 95% CI [1.28, 1.34]), and was highest when both parents were diagnosed (RR =1.52, 95% CI [1.46, 1.59]).
- For both the father and the mother, the risk increased in parents diagnosed with several different psychiatric disorders.
- The increased risk was present not only for children born preterm, but also for the larger group of offspring born at early term (37-38 weeks), who represent approximately 20% of all births.

What Do These Findings Mean?

- Psychiatric diagnoses in both parents are associated with gestational age.
- Whether additional social support and prenatal care to the families with a positive psychiatric history could have impact on gestational age requires further investigation.

INTRODUCTION

4. Line 64 – '…causes [1, 6, 7]…' please remove all spaces between citations for in-text reference callouts to read as follows '…causes [1,6,7]…'. Please check and amend throughout all sections of the manuscript including supporting files where relevant.

Reply:

We have made changes accordingly throughout the manuscript and supporting files.

5. Line 66 – 'systematical' suggest 'systematic'

Reply:

We have made changes accordingly (Line 72).

RESULTS

6. As above, please makes amendments at line 188 to improve clarity.

Reply:

Changes have been made accordingly in the Results (Line 196).

STATISTICAL REPORTING

7. In the abstract you use commas to separate upper and lower bounds (which we favour) and in the main manuscript results section, hyphens separate upper and lower bounds. Suggest using commas instead to prevent confusion with reporting of negative values. See line 208 onwards.

Reply:

Commas are used to separate upper and lower bounds instead of hyphen as suggested.

DISCUSSION

8. Line 276 – '…gestational age range…' instead perhaps Line 283 – suggest 'Only one study examined the risk of birth before full term. It included only mothers with psychiatric disorders but reported an increased risk of births before 39 weeks, consistent with our findings…' or similar for improved clarity/grammar.

Reply:
We have made changes accordingly at Line 283 and Line 292 in the revised version.

SOCIAL MEDIA

9. To help us extend the reach of your research, if not already done so, please detail any Twitter handles you wish to be included when we tweet this paper (including your own, your coauthors', your institution, funder, or lab) in the manuscript submission form when you re-submit the manuscript.

Reply:
Thank you. We have no additional Twitter handles to be included. We have mentioned this in the submission form.

We thank the editor for his/her additional comments.

Reviewer #1: Alex McConnachie, Statistical Review
I thank the authors for their consideration of my original comments.
I found their responses to be entirely satisfactory. I feel the changes made are for the better, and their reasons for not making changes in response to some comments are fully justified. I have no further comments to make.

Reviewer #2: The authors have adequately responded to my comments. I have nothing further.

We thank Dr. Alex McConnachie and the Reviewer #2 for their positive comments.

Reviewer #3
The has been improved substantially in line with the reviewers' comments. I have several minor substantive suggestions.
Reply: We thank the reviewer for the positive comments.

1. lines 50-52: It may be more informative to present the range of relative risks in each of the three exposure categories (fathers diagnosed, mothers diagnosed, both parents diagnosed) instead of or in addition to the average RR in each category.

Reply:
We have now presented the RR together with the range of RR in the Author Summary.
Line 53
"The risk of preterm birth consistently increased when fathers were diagnosed with different psychiatric disorders (relative risk (RR) =1.12, 95% confidence interval (CI) [1.08, 1.15]), increased further when mothers were diagnosed (RR =1.31, 95% CI [1.28, 1.34]), and was highest when both parents were diagnosed (RR =1.52, 95% CI [1.46, 1.59])."

2. 75-77: evidence suggests that advancing birth, even by as little as 1 or 2 weeks before full term, can affect neonatal morbidity [26] and future health outcomes [2]. The phrase "advancing birth" suggests an intervention to change the length of gestation, which speaks to one of the main challenges regarding preterm birth and shortened gestation in general, i.e., PTB is associated with adverse outcomes later on, but it's not clear whether interventions to prolong gestation would also reduce these other sequelae (as both PTB and later adverse outcome may stem from the same underlying problem). Accordingly, I would rephrase this sentence to clearly avoid causal language, e.g.: "evidence suggests that even moderately shorter gestation is associated with neonatal morbidity ..."

Reply: Thank you for the clarifying this concept for us. We have made changes as suggested.
Line 81
"Third, evidence suggests that even moderately shorter gestation, as 1 or 2 weeks before full term, is associated with neonatal morbidity...."

3. Line 175: before two years of conception - I recommend "prior to two years before conception". Similarly, at line 238: "psychiatric diagnoses first diagnosed at least two years before conception"

Reply: We adjusted accordingly (Line 183 and Line 247).

4. 176: to see how much - change to "To determine the extent to which the results were driven by individuals with provisional diagnoses"

Reply: We adjusted accordingly Line (185).

5. 89-90: I suggest "parents born in Sweden, Finland, Norway, Denmark or Iceland"

Reply: We adjusted accordingly (Line 95).

6. 193 and elsewhere - "25th to 75th percentiles" can be changed to "interquartile range" (or "IQR")

Reply: We have made changes throughout the manuscript. Thanks for the suggestion.

7. 197: was shown in Figure C in S1 Appendix - change to "is shown..."

Reply: We adjusted accordingly (Line 205).

8. 13-14: We extended the analysis beyond the PTB - I suggest "... beyond PTB"

Reply: We adjusted accordingly (Line 13).

9. 25-26: Please report which disorders the 3 different estimates correspond to (as you do in the main text, lines 217-219).

Reply: We adjusted accordingly (Line 26).

10. 49-50: when fathers were diagnosed of different psychiatric disorders - I suggest "... diagnosed with different psychiatric disorders"

Reply: We adjusted accordingly (Line 53).

11. 59: could have impact on gestational age - I suggest "could have an impact ..."

Reply: We adjusted accordingly (Line 65).

12. 66: One recent systematical review - change to "... systematic review"

Reply: We adjusted accordingly (Line 72).

13. 73-74: a rigorous study addressing the combined effect of psychiatric disorders in the couples is notably absent - I suggest "a rigorous study addressing the combined effect of psychiatric disorders in couples ..."

Reply: We adjusted accordingly (Line 79).

14. 91: The fathers were identified - I suggest "Fathers were identified"

Reply: We adjusted accordingly (Line 97).

15. 94: Psychiatric diagnoses from specialist care were extracted in the Swedish National Patient Register (NPR) - "... extracted from ..."

Reply: We adjusted accordingly (Line 100).

16. 97: The data quality of the NPR has been verified [29] , and validated - remove comma before "and validated"

Reply: We adjusted accordingly (Line 103).

17. 100-101: European Unions Horizon 2020 research and innovation program - change to "European Union's Horizon 2020 research and innovation program"

Reply: We adjusted accordingly (Line 109).

18. 135-136: Where a linear regression is analysing differences in the mean between exposures - I suggest "Whereas a linear regression analyses differences in the mean between exposures"

Reply: We adjusted accordingly (Line 144).

19. 139: the quantile regression can estimate at which gestational age - remove "the"

Reply: We adjusted accordingly (Line 148).

20. 145-146: Then we examined the risk by six psychiatric diagnostic categories and by subtypes under the categories. I suggest "We then examined the risk by six psychiatric diagnostic categories and by subtypes within each category."

Reply: We adjusted accordingly (Line 154).

21. 194 and 195: change "2 year" to "2 years"

Reply: We adjusted accordingly (Line 203).

22. 205: typo for "gestational age"

Reply: We adjusted accordingly (Line 214).

23. 244: spanning over 20 years, and including 1.5 million births - I would remove the comma before "and"

Reply: We adjusted accordingly (Line 253).

24. 252-253: the risk was largely similar for maternal associations, but noticeably reduced for paternal associations, although remained statistically significant. I suggest "the risk was largely similar for maternal associations but was noticeably reduced for paternal associations, although it remained statistically significant."

Reply: We adjusted accordingly (Line 261).

25. 257-278: gestational age determined using ultra sound - "ultrasound" 1 word (as at line 113)

Reply: We adjusted accordingly (Line 266).

26. 258-259: a publicly financed health care system with equal access - I suggest "... with universal access"

Reply: We adjusted accordingly (Line 268).

27. 264-265: Although we have examined the risk by diagnostic categories and subtypes of psychiatric disorders - I suggest "Although we examined ..."

Reply: We adjusted accordingly (Line 273).

28. 302-303: Increased psychological stress has been linked to higher levels of stress hormones as cortisol which may induce premature uterus contractions - I suggest "... higher levels of stress hormones such as cortisol, which may induce premature uterine contractions"

Reply: We adjusted accordingly (Line 312).

29. 318-319: it is not clear if these are associated also with psychiatric conditions - I suggest "it is not clear if these are also associated with psychiatric conditions"

Reply: We adjusted accordingly (Line 327).

30. 330-331: However, such psychological support is probably lacking in which both of the couples have psychiatric conditions. Change "in which both of the couples" to "in which both members of the couple". I would also consider changing "is probably lacking" to "may be lacking" to highlight the somewhat speculative nature of the statement and avoid an accusatory tone.

Reply: We adjusted accordingly (Line 340).

31. 335-336: whether additional social support to these families is need - I would change "is need" to "may be needed"

Reply: We adjusted accordingly (Line 346).

32. 338-339: However, risk from downstream factors, e.g., obesity and smoking, as well as public health and intervention may differ between countries and populations. The phrase "public health and intervention" is unclear. Should this perhaps be something like "public health initiatives" or "public health interventions"?

Reply: We have made changes and used the phrase ‘public health interventions’ instead (Line 349).

Page 13, line 4: Remove the comma after "Sweden" and add a comma after "psychiatric diagnosis(es)"
Reply: We adjusted accordingly (Line 337).

We thank the Reviewer 3 for his/her patient additional comments and editing.
